# Peptide-Based Capture of Chikungunya Virus E2 Protein Using Porous Silicon Biosensor

**DOI:** 10.3390/s21248248

**Published:** 2021-12-10

**Authors:** Rabeb Layouni, Tengfei Cao, Matthew B. Coppock, Paul E. Laibinis, Sharon M. Weiss

**Affiliations:** 1 Department of Chemical & Biomolecular Engineering, Vanderbilt University, Nashville, TN 37235, USA; rabeb.layouni@vanderbilt.edu (R.L.); paul.e.laibinis@vanderbilt.edu (P.E.L.); 2 Interdisciplinary Material Science Program, Vanderbilt University, Nashville, TN 37235, USA; tengfei.cao@vanderbilt.edu; 3 Human Research and Engineering Directorate, DEVCOM Army Research Laboratory, Adelphi, MD 20783, USA; matthew.b.coppock.civ@army.mil; 4 Department of Electrical and Computer Engineering, Vanderbilt University, Nashville, TN 37235, USA

**Keywords:** peptide, optical biosensor, Chikungunya virus, porous silicon

## Abstract

The detection of pathogens presents specific challenges in ensuring that biosensors remain operable despite exposure to elevated temperatures or other extreme conditions. The most vulnerable component of a biosensor is typically the bioreceptor. Accordingly, the robustness of peptides as bioreceptors offers improved stability and reliability toward harsh environments compared to monoclonal antibodies that may lose their ability to bind target molecules after such exposures. Here, we demonstrate peptide-based capture of the Chikungunya virus E2 protein in a porous silicon microcavity biosensor at room temperature and after exposure of the peptide-functionalized biosensor to high temperature. Contact angle measurements, attenuated total reflectance—Fourier transform infrared spectra, and optical reflectance measurements confirm peptide functionalization and selective E2 protein capture. This work opens the door for other pathogenic biomarker detection using peptide-based capture agents on porous silicon and other surface-based sensor platforms.

## 1. Introduction

Porous silicon (PSi) has gained significant attention over the past two decades in part due to its capability to serve as a highly sensitive material platform for optical biosensing [1,2,3,4,5,6]. PSi-based optical biosensors have been demonstrated for the detection of a variety of target molecules including oligonucleotides [7,8], proteins [9,10,11], and enzymes [12,13]. Many architectures have been employed for PSi optical biosensors, ranging from simple single-layer interferometers to resonant multilayer microcavities and two-layer on-chip ring resonators [6,14,15]. Moreover, PSi optical biosensors have been shown to be compatible with both benchtop lab-on-chip systems and smartphone-based detection schemes [16,17,18,19]. A key advantage of PSi is its large surface area, which enables the immobilization of a high concentration of bioreceptors for capturing target molecules of interest. Due to the wide range of surface modifications that can be supported on PSi, several different types of bioreceptors have been utilized in PSi optical biosensors, including oligonucleotides [20,21], aptamers [22,23], and antibodies [24,25]. The choice of bioreceptor to employ depends strongly on the application, as there is often a tradeoff between selectivity, affinity, and robustness.

Across all sensor platforms, the current gold standard among bioreceptors are monoclonal antibodies (mAbs), which exhibit high affinity and excellent selectivity [26]. However, their poor in vitro adaptability, manufacturability, and stability during transportation and storage greatly restrict their applications, especially for point-of-care (POC) applications in operational environments. To address some of these challenges, alternative capture agents have been developed such as DNA (or RNA) aptamers [27], single domain antibodies [28], post-modified antibodies, and PNAs [29]. Unfortunately, none of these bioreceptors have succeeded in overcoming all stability issues.

Peptides, which are short chains of amino acids and the building blocks of proteins, have emerged as a promising capture agent for POC testing in harsh environments. Although smaller in size than mAbs, peptides (and their derivatives) have shown comparable affinity and selectivity for certain targets [30,31,32]. Like antibodies, peptides have the advantage of mature screening protocols, diverse structures, and large chemical versatility. Furthermore, they offer on-demand selection and synthesis, excellent thermal stability, and good adaptability to various platforms through chemical modifications [30]. Peptide-based capture agents have been demonstrated in a variety of biosensor platforms including electrochemical biosensors [33], nanowire-based sensors [34], colorimetric assays [35], and fluorescence-based sensors [32]. Beyond their function as bioreceptors, peptide sequences have also been exploited for their selective affinity to semiconductor surfaces, one of the common transducing materials for biosensors [36,37].

In this work, we combine the advantages of robust peptide-based bioreceptors with the versatile PSi optical biosensor platform to demonstrate its detection of the Chikungunya virus E2 protein including after exposure of the sensor to an elevated temperature prior to incubation. We leverage our prior work developing a protein catalyzed capture (PCC) agent for E2 [32] and establishing a methodology for immobilizing the PCC agents on PSi [31]. Contact angle, Fourier transform infrared (FTIR) spectroscopy, and reflectance measurements are performed to characterize the PSi biosensor during functionalization and confirm and quantify E2 protein capture. Advanced understanding of the peptide−PSi interface provides critical insights that could be applied to peptide-modification of other nanostructured surfaces, leading to improved performance across multiple sensing technologies.

## 2. Materials and Methods

### 2.1. Materials

Single-side polished, boron-doped p-type silicon wafers (⟨100⟩, 0.01–0.02 Ω cm, 500–550 μm) were purchased from Pure Wafer, WRS Materials Company. Hydrofluoric acid (HF) was purchased from Acros Organics. 1,8-nonadiyne, 97%, was purchased from Alfa Aesar. The E2-binding peptide was synthesized as described in Section 2.4. Recombinant, non-glycosylated Chikungunya virus E2 protein was purchased from ImmunoDX, LLC.

### 2.2. Porous Silicon Microcavity Fabrication

PSi microcavities (PSiMs) are fabricated by electrochemical etching of p-type (0.01 Ω⋅cm) silicon wafers in a 15% hydrofluoric acid (HF) solution in ethanol (EtOH), in a similar manner to what we reported previously [16]. A sacrificial layer of PSi is first etched at a current density of 70 mA/cm^2^ for 100 s and is subsequently removed with a NaOH solution (1:9 1 M NaOH to EtOH). Secondly, the PSiM structure is fabricated by applying the following current density profile: alternating current densities of 80 mA/cm^2^ for 3.1 s and 60 mA/cm^2^ for 3.2 s repeated seven times, followed by 80 mA/cm^2^ for 6.2 s, and then another set of seven repeats of alternating current densities of 60 mA/cm^2^ for 3.2 s and 80 mA/cm^2^ for 3.1 s. This traditional one-dimensional thin film microcavity structure comprises two multilayer Bragg mirrors separated by a cavity layer.

### 2.3. Thermal Hydrosilylation

The surfaces of the PSiM were passivated by thermal hydrosilylation with 1,8-nonadiyne. Prior to the hydrosilylation reaction, nonadiyne underwent three freeze−pump−thaw cycles for degassing, and the native oxide on the as-anodized PSiM was removed by dipping samples in a 2.5% HF solution (aq), followed by washing with DI water and ethanol. Samples were thoroughly blown dry with nitrogen before insertion into a Schlenk flask. The hydrosilylation reaction was conducted over 3 h by heating the Schlenk flask in an oil bath at 155 °C. After allowing the reaction solution to cool to room temperature, the PSi sample was removed from the flask, soaked in dichloromethane, rinsed with ethanol, and then dried with nitrogen.

### 2.4. Peptide Synthesis and Attachment

The E2-binding cyclic peptide (cy(YWHWS)) was synthesized and screened following previously established protocols [32]. Briefly, the linear peptide Lys(N_3_)-YWHWS-Pra was first synthesized on Rink amide resin using conventional Fmoc-based solid phase synthesis in a Biotage Initiator+ Alstra microwave synthesizer. The peptide was cyclized using copper(I) iodide (1.5 equiv.) and ascorbic acid (5 equiv.) in 4:1 NMP:piperidine overnight at room temperature. The residual copper was removed by shaking the resin with 5% (*w*/*v*) sodium diethyldithiocarbamate trihydrate and 5% (*v*/*v*) *N*,*N*-diisopropylamine in 1-methyl-2-pyrrolidinone for 8 min (2x). Lys(N_3_) was then coupled to the N-terminus and Fmoc was removed. The peptide was cleaved from the resin for 2 h with 87.5% trifluoroacetic acid (TFA), 5% water, 5% phenol, and 2.5% triisopropylsilane (TIPS), and then purified by reverse phase HPLC using a C_18_ column. The mass (cy(YWHWS) + H^+^) was confirmed by MALDI-TOF (calculated: 1181.3 *m*/*z*; actual: 1181.14 *m*/*z*; Figure S1 in Supplementary Materials).

The attachment of the E2-binding peptide to the 1,8-nonadiyne modified PSiM was achieved by copper(I)-catalyzed azide alkyne cycloaddition, following a procedure similar to that used to click streptavidin-binding peptide to PSi [31]. A mixture of 60 μL azide-modified E2-binding peptide (8.67 mM, ethanol/water 1:1), 20 µL copper (II) sulphate pentahydrate (0.40 mM in water), and 20 μL sodium ascorbate (20.19 mM in water) was added to the alkyne-terminated PSiM samples. After 24 h, PSi samples were rinsed with anhydrous ethanol and DI water followed by exposure to a 0.5 mM HCl solution for 30 min to remove residual copper. Samples were rinsed with DI water and anhydrous ethanol and then dried with nitrogen.

### 2.5. E2 Protein Sensing

The ability of the surface-bound E2-binding peptide to capture the target E2 protein was investigated through exposure of the peptide-modified PSiM to E2 protein for 3 h. Due to the hydrophobicity of the peptide-modified PSi, vacuum-assisted infiltration was carried out. First, the peptide-modified PSi sample was placed in the bottom of a 25 mL filtering flask that was sealed with a rubber stopper. A vacuum line was connected, and the flask was evacuated to <10 Torr. Next, a 50 μL volume of 1 μM E2 protein in water was carefully drop-cast onto the PSi sample using a syringe with a 6-inch-long needle that penetrated the rubber stopper. The vacuum was subsequently released slowly to atmospheric pressure, promoting infiltration of the E2 protein solution into the PSi pores. The PSi sample was incubated with the E2 protein solution for 3 h, then removed from the flask, rinsed with DI water, and dried with nitrogen. This vacuum-assisted infiltration process was repeated for three PSiMs. An additional PSiM was incubated in a 50 μL volume of 1 μM chicken ovalbumin in water using the vacuum-assisted process, serving as a control. All PSi samples were evaluated with FTIR spectroscopy and optical reflectance measurements.

### 2.6. Heat Treatment of Peptide Capture Agent

To verify the stability of the E2-binding peptide capture agent under high temperature when immobilized on PSi, peptide-functionalized PSi samples were stored in a furnace at 90 °C for 2 h. E2 sensing, as described in Section 2.4, was performed immediately afterward to confirm that the affinity of the E2-binding peptide was not affected by the exposure to high temperature.

### 2.7. Optical Measurements

Reflectance measurements were performed using a Newport Oriel 6000 Q Series lamp and fiber-coupled Ocean Optics USB-4000 spectrometer. The reflectance spectrum of a single-mode PSiM, such as those utilized in this work, is characterized by a spectral region of high reflectance and a single resonance within this spectral region. In the context of PSi-based biosensors, observed spectral shifts to longer wavelengths (i.e., redshifts) suggest the attachment and/or capture of biomolecules inside the pores of the PSi sample. On the other hand, spectral shifts to shorter wavelengths (i.e., blueshifts) often suggest removal of material or the possibility of corrosion of the PSi film [21]. Four samples were prepared and measured for each set of conditions investigated.

### 2.8. Water Contact Angle Measurements

Measurements were performed on sessile drops (~5 microliter) of DI water on PSiM sample surfaces using an optical tensiometer (T114, Attension). For each set of conditions, two samples were measured.

### 2.9. ATR-FTIR Measurements

Attenuated total reflection Fourier transform infrared (ATR-FTIR) spectra of the PSiM samples were obtained with a Bruker Tensor 27 FTIR in a reflection mode from 400 to 4000 cm^−1^ at a resolution of 4 cm^−1^. Each measurement was averaged over 256 scans.

## 3. Results and Discussion

**Figure 1:**
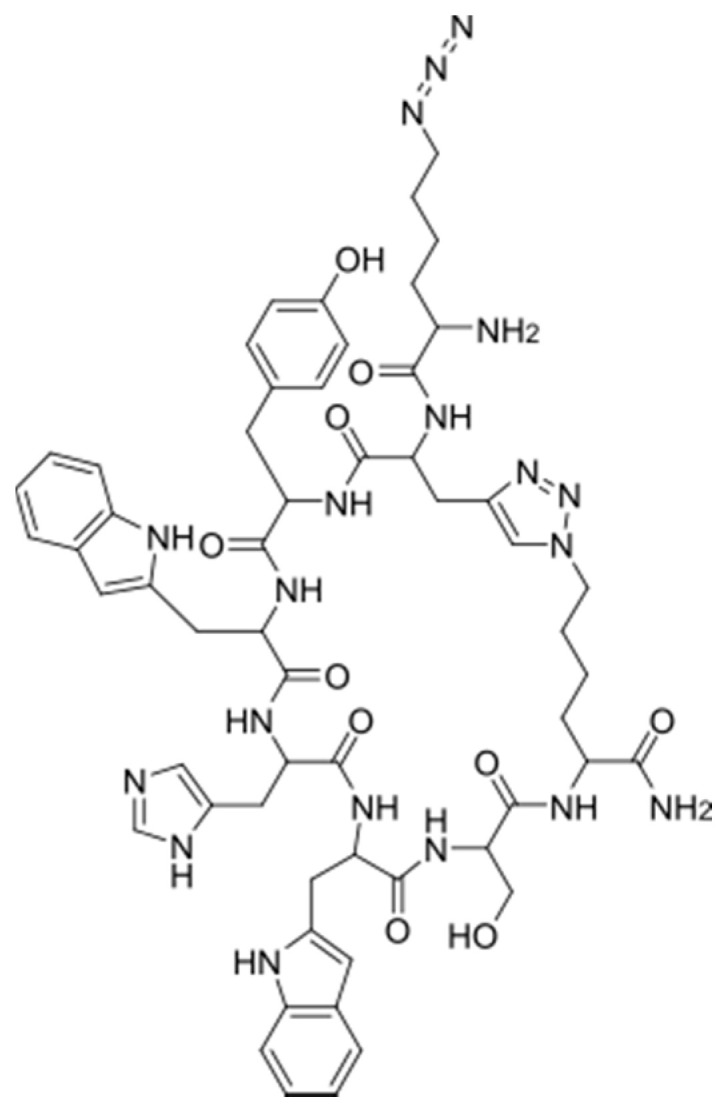
Structure of azide-modified E2-binding peptide.

**Figure 2:**
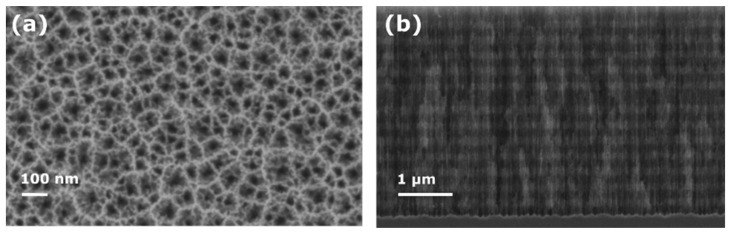
Porous silicon microcavity. (**a**) Top view and (**b**) cross-sectional view SEM images of PSi microcavity.

The PSi sensors used in this work were prepared in the following manner. First, PSi multilayer microcavities were formed by electrochemical etching. Thermal hydrosilylation was then performed to attach 1,8-nonadiyne (ND) to the pore walls. Next, the azide-modified E2-binding peptide (Figure 1) was immobilized on the alkyne-modified PSi surfaces using a copper-catalyzed click reaction. We opted for click chemistry over other coupling methods for its ability to attach any peptide sequence without concern of potential side reactions with amino acid residues in the peptide. Finally, the target E2 protein was exposed to the peptide-functionalized PSiMs. Figure 2a,b shows scanning electron microscopy (SEM) images of a typical as-anodized PSiM. The two seven-period Bragg mirrors and central cavity layer of the PSiM can be seen from the cross-sectional SEM image (Figure 2b), with the darker layers corresponding to higher porosity layers and the brighter layers corresponding to lower porosity layers. The average pore diameters in the high and low porosity layers are approximately 60 and 40 nm, respectively. We note that there is a pore size distribution in each layer, which can be seen in the top-view SEM image in Figure 2a.

### 3.1. Contact Angle Measurements

**Figure 3:**
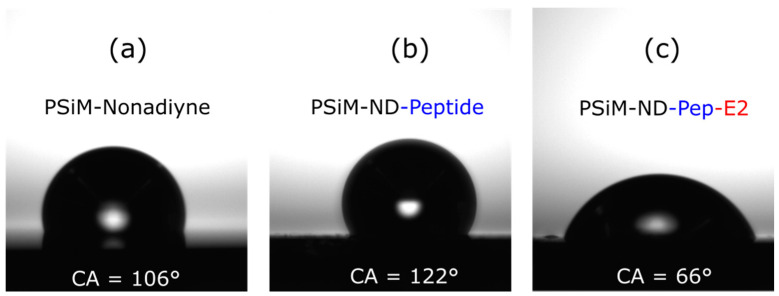
Surface wettability. Water droplets and their contact angles on (**a**) nonadiyne (ND)-modified PSi microcavity (PSiM); (**b**) peptide-ND-modified PSiM; and (**c**) peptide-ND-modified PSiM after E2 protein capture. Water contact angles greater than 90° after ND and peptide attachment indicate a hydrophobic surface that can hinder wetting of the PSi surface, which therefore impedes molecular diffusion into the pores. The lower contact angle after E2 protein exposure indicates that the PSi surface became hydrophilic and is further confirmation of the successful capture of the E2 protein by the PSi sensor.

Contact angle measurements were taken after each preparation step to investigate surface wettability. Freshly etched PSi is innately hydrophobic due to the presence of silicon hydrides on its surface as well as the trapping of air within its porous structure. The water contact angle on the freshly etched PSiM was ~134° (avg = 130 ± 6°, *N* = 2) (see Figure S2 in Supplementary Materials). Subsequent surface modification alters the contact angle based on the wettability of the coating material. As shown in Figure 3a, after ND thermal hydrosilylation, the water contact angle was ~106° (avg = 105 ± 10°, *N* = 2). This high water contact angle on the PSi silicon surface suggests a good alkyne surface coverage following the attachment of 1,8-nonadiyne. Upon clicking the capturing peptide for E2 to the PSi surface, the water contact angle increased to ~122° (avg = 120 ± 4°, *N* = 2) (Figure 2b). Unfortunately, this low surface wettability inhibits the delivery of target protein from an applied aqueous solution into the pores as needed for sensing. As the peptide capture agents identified to have high affinity to the E2 protein generally include multiple hydrophobic amino acids [32], we expect that similar results as in Figure 2b would occur using other E2-capturing peptides. To mitigate this surface wettability issue, we modified our usual experimental protocol by including a vacuum-assisted technique to facilitate improved infiltration of the target E2 solution into the pores. After exposure of the sensor to the E2 protein by this approach, the contact angle on the PSi sample decreased to ~66° (avg = 68 ± 3°, *N* = 2), suggesting successful E2 capture with the surface becoming more hydrophilic from protein attachment in the pores. Optical reflectance measurements discussed in Section 3.3 confirm the specific capture of the E2 protein. In future work, we anticipate that the use of an alternative sensor architecture, such as a flow-through membrane in a microfluidic device, can mitigate the aforementioned transport challenges without the need for the vacuum assistance [38].

### 3.2. ATR-FTIR Measurements

**Figure 4:**
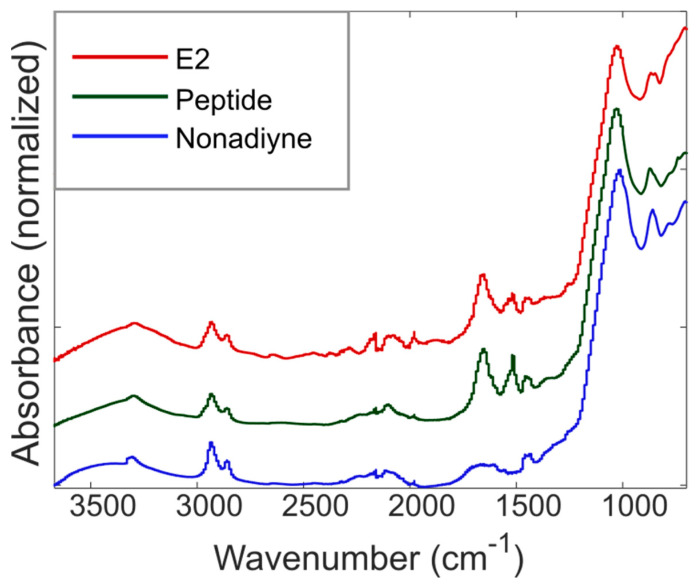
Surface functionalization and ATR-FTIR absorbance spectra. ATR-FTIR spectra confirming the attachment of the desired materials as described in the main text. A sharp peak near 3320 cm^−1^ and a weak peak at 2100–2140 cm^−1^ indicate the presence of alkyne groups associated with the nonadiyne. Furthermore, strong peaks at 2860 and 2930 cm^−1^ corresponding to C-H stretching and a weak peak at 1668–1687 cm^−1^ indicating C=C stretching are observed. Upon click reaction with the azide-modified peptide, the peak near 3320 cm^−1^ reduces in intensity, indicating partial reaction of the alkyne groups to attach the peptide capture agent by a 1,2,3-triazole. Amide I and amide II bands are also observed near 1650 and 1520 cm^−1^, respectively, confirming the presence of attached peptide molecules. After capture of the E2 protein, the spectra include peaks associated with amino acids in the peptide and E2 protein.

While the water contact angle measurements provide strong indirect evidence of the desired surface functionalization and target protein capture in the PSi sensors, ATR-FTIR measurements reveal direct evidence of the molecular attachments during functionalization via observation of absorption peaks that correspond to molecular vibrations of constituent chemical bonds. Figure 4 shows ATR-FTIR spectra obtained after each functionalization step and after exposure of the sensor to the E2 target protein. A peak near 3320 cm^−1^ and a weak peak at 2100–2140 cm^−1^ indicate the presence of the alkyne group associated with nonadiyne. In addition, strong peaks at 2860 and 2930 cm^−1^ corresponding to C-H stretching and a weak peak near 1668–1687 cm^−1^ indicating C=C stretching provide additional support of nonadiyne attachment. It is worth noting that sharp silicon hydride (Si-H) peaks near 2100 cm^−1^ observed for freshly etched PSi (shown in Figure S2 in Supplementary Materials) are absent due to the consumption of Si-H during the nonadiyne hydrosilylation reaction. After the click reaction with the azide-peptide molecule, there are two primary changes in the ATR-FTIR spectrum. First, the intensity of the peaks corresponding to the alkyne groups shows a decrease due to transformation of these groups during the click reaction. Second, the appearance of amide bands near 1650 and 1520 cm^−1^ further confirms the attachment of the peptide capture agent. We note that the persistence of the peak at 3320 cm^−1^ after peptide attachment, albeit attenuated, suggests that the conversion during the click reaction was not high and that many alkyne groups remained unreacted. Accordingly, peptide surface coverage may be improved by utilizing a longer click reaction time and/or an increase in the starting concentration of the azide-modified peptide. After incubation, amide band peaks associated with amino acids in the E2 protein are observed as well, but the changes are slight. Our interpretation is that the IR spectrum is compatible with the presence of both the capture agent and the E2 protein but does not confirm binding of the latter. We note that the IR spectrum after E2 exposure is consistent with the wetting and reflectance data in Section 3.1 and Section 3.3 that provide strong support of E2 binding to the capture agent. Approaches to improve transport of E2 protein into the PSIM are likely to increase binding, and these are being pursued. Overall, the surface modification method utilized in this work is straightforward, scalable, and can be applied to other sensing applications where biomolecules can be clicked onto a surface.

### 3.3. Reflectance Measurements

**Figure 5:**
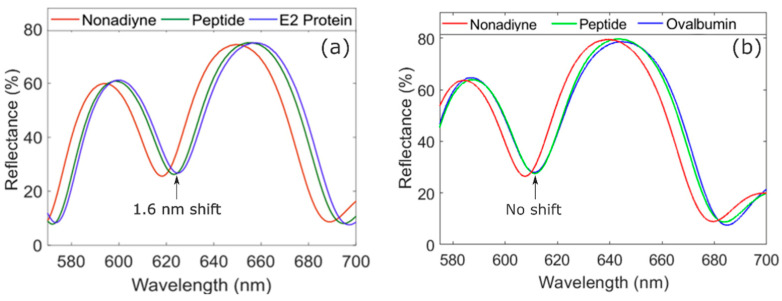
Reflectance measurements from E2 sensing and control experiments. (**a**) Reflectance spectra showing spectral redshift after clicking the peptide capture agent to the alkyne-terminated PSi sensor and an additional 1.6 nm spectral redshift after exposure of the peptide-modified PSi sensor to 1 µM E2 protein in DI water. The spectral redshifts confirm peptide functionalization and target E2 protein capture and detection. (**b**) Reflectance spectra for control experiment showing spectral redshift after peptide functionalization of the PSi sensor and no spectral shift after exposure of the sensor to 1 µM non-target chicken ovalbumin in water, which confirms the specificity of the peptide capture agent.

As further evidence of molecular attachment during functionalization and E2 protein capture in the peptide-modified PSi sensors, reflectance measurements were performed after each functionalization step and after exposure of the sensor to E2 protein. The reflectance spectrum of a PSiM exhibits a characteristic resonance, as shown in Figure 5. The resonance wavelength is highly sensitive to the optical thickness of the PSiM, particularly that of the cavity layer. When molecules attach in the PSiM, the effective refractive index and therefore the optical thickness of the PSi layers in which the molecules are bound increases. This increase in optical thickness results in a redshift of the resonance wavelength. Resonance redshifts in a representative sample can be seen in Figure 5a after peptide modification and after exposure of the PSi sensor to E2 protein at room temperature. The larger magnitude of the resonance shift after peptide attachment (avg = 6.4 ± 2 nm, *N* = 4) compared to that after E2 capture (avg = 1.7 ± 0.3 nm, *N* = 4), combined with the fact that the peptide has a smaller molecular weight than the E2 protein, suggests that more peptides are immobilized in the PSiM than the number of E2 proteins that are captured. Hence, it is likely that the described protein infiltration challenges due to the hydrophobicity of the peptide-modified PSi inhibit more efficient target capture. In addition, mass transport limitations of the protein, due to the finite size of the pores, may also impede efficient E2 infiltration and capture. As mentioned earlier, an alternative PSi sensor architecture such as a flow-through microcavity membrane [38] is likely to facilitate improved E2 infiltration, and complementary techniques such as mixing during target incubation [39] can promote improved mass transport of the target molecules. Both are expected to offer improvement here. In addition, alternative peptide immobilization strategies could be explored to create a more hydrophilic surface and increase surface wettability; however, such surfaces may be susceptible to non-specific binding, and care must be taken to ensure there is sufficient surface passivation to prevent corrosion of the PSi matrix upon exposure to aqueous solutions [21].

To exclude the possibility of non-specific binding by the E2 protein and artifacts of the vacuum-assisted infiltration process causing the resonance redshift of the PSi sensor upon exposure to the E2 target protein, we performed a control experiment where we exposed the peptide-modified PSi sensor to a similarly sized non-target protein (chicken ovalbumin) using the same experimental protocols as for the E2 detection experiment. As shown in Figure 5b, the resonance redshift (avg = 5.8 ± 3.8 nm, *N* = 4) in a representative sample after peptide modification in this control experiment confirms that the E2-binding peptide capture agent is present in the PSi sensor in a quantity similar to that in the PSi sensors exposed to target E2 protein. However, unlike the case for E2 protein capture, there is a negligible resonance shift after exposure of the peptide-modified PSi sensor to chicken ovalbumin protein. Hence, we can conclude that the E2-binding peptide retains its specificity when immobilized on the PSi surface. We further note that a negligible resonance shift was also measured in additional control experiments in which the E2 protein was exposed to nonadiyne-modified PSiMs without the E2-binding peptide capture agent present on the surface.

### 3.4. High-Temperature Experiment

**Figure 6:**
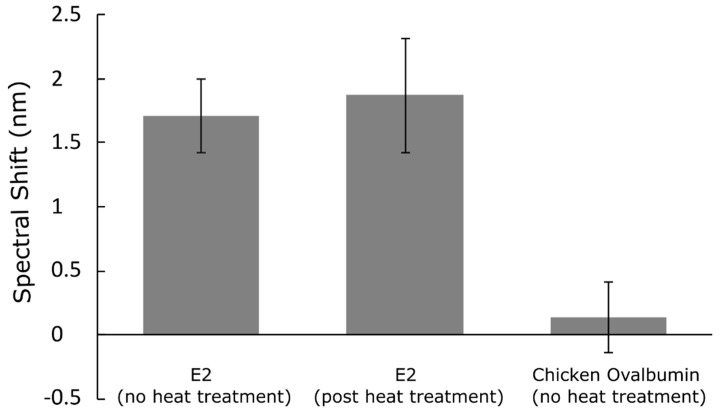
Detection of E2 protein after exposure to high-temperature environment. Comparison of spectral resonance shift of PSi sensor after capture of E2 protein under normal conditions (i.e., without exposure to elevated temperature) and after the PSi sensor was exposed to high temperature (90 °C) immediately prior to incubation in the E2 protein solution. Similar magnitude redshifts (1.7 ± 0.3 nm compared to 1.9 ± 0.5 nm, *N* = 4) are observed for both conditions, which demonstrates the robustness, stability, and preserved biofunctionality of the peptide capture agents in harsh conditions. In contrast, a negligible spectral shift (0.1 ± 0.3 nm, *N* = 4) is observed after the control solution of chicken ovalbumin is exposed to the PSi sensor, confirming the specificity of the E2-binding peptide.

We tested the stability and binding activity of the immobilized E2-binding peptide in the PSi sensor after its exposure to a temperature well above those that might be encountered when sensors are deployed in operational conditions. Here, we repeated the E2 sensing experiments after peptide-modified PSi sensors were subjected to a 90 °C ambient for 2 h. Prior work has shown that anti-E2 antibodies do not maintain high affinity for target E2 protein after being heated at 90 °C for 1 h, while E2-binding peptide retains its binding activity after such a heat treatment; we note that this prior work was conducted using a bead-based Luminex assay and not while the capture agents were immobilized on a nanostructured surface [32]. Figure 6 summarizes the reflectance measurement results of the room temperature and high-temperature E2 sensing experiment. Similar spectral shifts are measured with and without the heat treatment when the peptide-modified PSi sensors are exposed to target E2 protein. Thus, the E2-binding peptide capture agents retain their binding activity in the PSi sensors after exposure to the high temperature. This robustness and stability of the E2-binding peptide makes it a strategic practical choice for the detection of the Chikungunya virus using nanostructured sensor material platforms.

## 4. Conclusions

Peptide-based capture of Chikungunya virus E2 protein was demonstrated on a PSi sensor platform. Water contact angle, ATR-FTIR spectroscopy, and reflectance measurements all confirm the desired functionalization of the PSi microcavities with E2-binding peptide and the successful capture of the target E2 protein. Importantly, the peptide-modified PSi sensor performed similarly for E2 protein capture after storage at room temperature and after exposure of the sensor to an elevated temperature of 90 °C for 2 h. The stability of the E2-binding peptide capture agents immobilized in the nanostructured pores of the PSi sensor suggest not only that these peptides are an excellent choice for the detection of Chikungunya virus in harsh environments, but also that peptide-based capture agents should continue to be explored more broadly for sensing done in the field and outside a traditional laboratory environment.

## Data Availability

Data underlying the results presented in this paper are not publicly available at this time but may be obtained from the authors upon reasonable request.

## Supplementary Materials

The following are available online at https://www.mdpi.com/article/10.3390/s21248248/s1, Figure S1: Mass spectrum of Lys(N_3_)-Cy(YWHWS), Figure S2: Contact angle measurement and ATR-FTIR spectrum of neat PSiM.
