# Supplementary material for: Peptide-Based Capture of Chikungunya Virus E2 Protein Using Porous Silicon Biosensor
Source: Sensors (Basel). 2021 Dec 10;21(24):8248. doi: 10.3390/s21248248 (PMC8708774; doi:10.3390/s21248248)
Supplement: Supplementary file 1 [file sensors-21-08248-s001.zip › sensors-1479399-supplementary.pdf]

Supplementary Materials

Peptide-Based Capture of Chikungunya Virus E2 Protein Using Porous Silicon Biosensor

Rabeb Layouni [1], Tengfei Cao [2], Matthew B. Coppock [3], Paul E. Laibinis [1,2] and Sharon M. Weiss [2,4,*]

[1] Department of Chemical & Biomolecular Engineering, Vanderbilt University, Nashville, TN 37235, USA; rabeb.layouni@vanderbilt.edu (R.L.); paul.e.laibinis@vanderbilt.edu (P.E.L.)
[2] Interdisciplinary Material Science Program, Vanderbilt University, Nashville, TN 37235, USA; tengfei.cao@vanderbilt.edu
[3] Human Research and Engineering Directorate, DEVCOM Army Research Laboratory, Adelphi, MD 20783, USA; matthew.b.coppock.civ@army.mil
[4] Department of Electrical and Computer Engineering, Vanderbilt University, Nashville, TN 37235, USA
* Correspondence: sharon.weiss@vanderbilt.edu

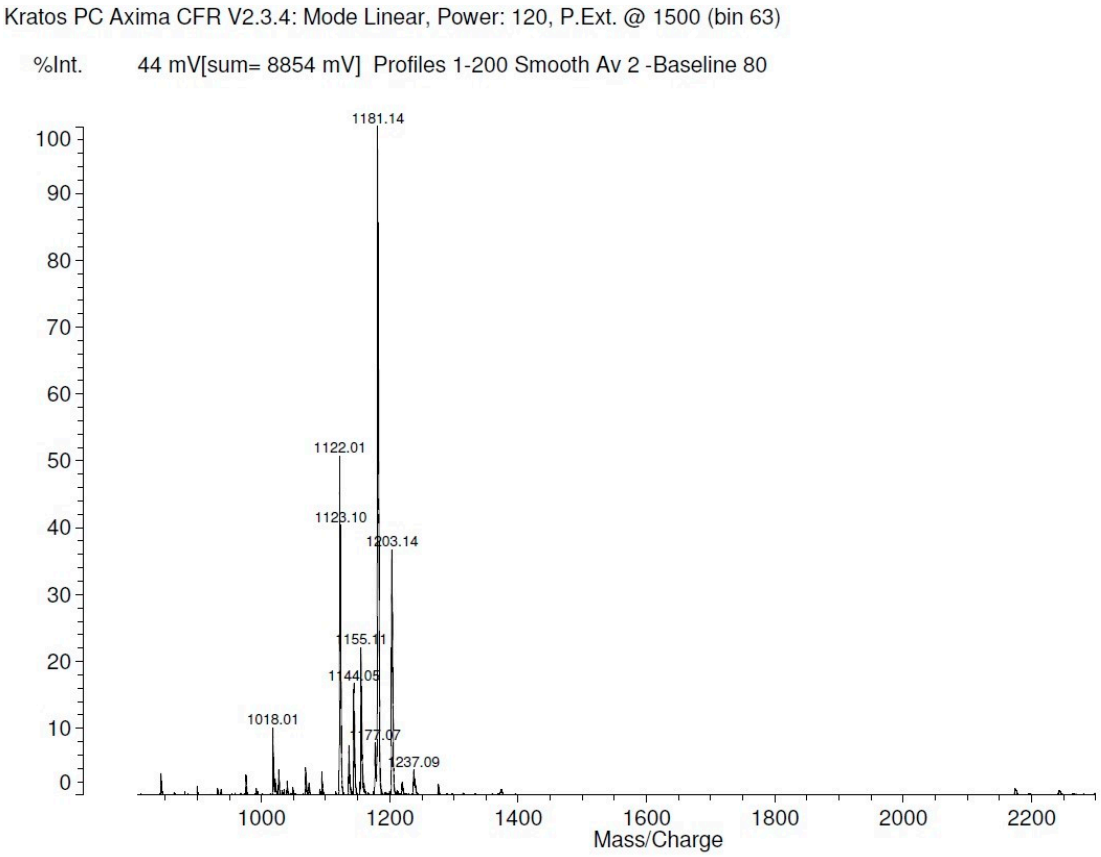

Figure S1. Mass spectrum of Lys(N_3)-Cy(YWHWS). MALDI-TOF MS (m/z): calcd (M+H) 1181.3; found 1181.14.

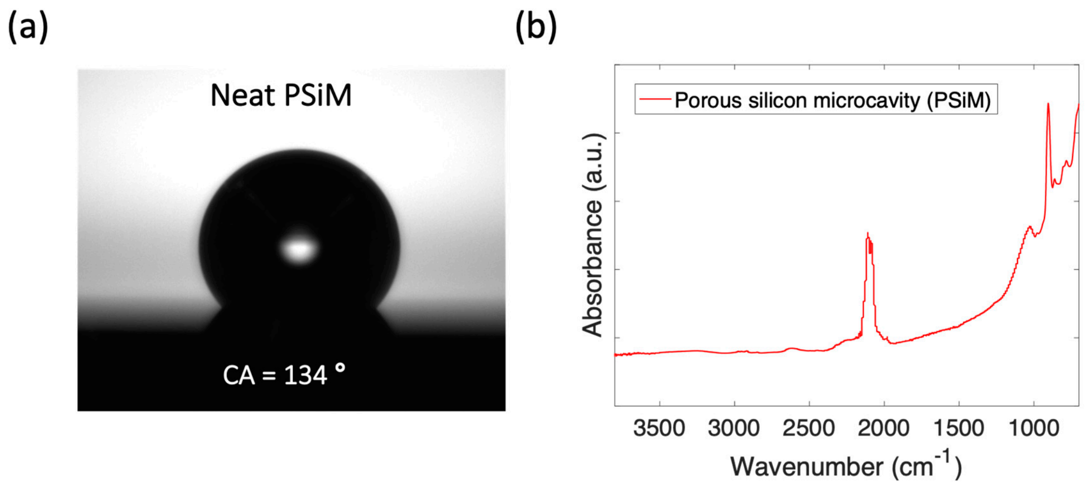

Figure S2. Neat porous silicon microcavity (PSiM) characterization. (**a**) Water contact angle measurement on freshly etched PSiM. The contact angle of ~134° (avg = 130° ± 6°, N = 2) indicates a hydrophobic surface. (**b**) ATR-FTIR absorbance spectrum of freshly etched PSiM. The sharp peaks near 2100 cm^{-1} are characteristic of silicon hydride (Si-H) bonds.
